# Detection and clinical significance of *CEACAM5* methylation in colorectal cancer patients

**DOI:** 10.1111/cas.16012

**Published:** 2023-11-09

**Authors:** Sheng‐Chieh Huang, Shih‐Ching Chang, Tsai‐Tsen Liao, Muh‐Hwa Yang

**Affiliations:** ^1^ Institute of Clinical Medicine National Yang Ming Chiao Tung University Taipei Taiwan; ^2^ Division of Colorectal Surgery, Department of Surgery Taipei Veterans General Hospital Taipei Taiwan; ^3^ Faculty of Medicine, School of Medicine National Yang Ming Chiao Tung University Taipei Taiwan; ^4^ Graduate Institute of Medical Sciences, College of Medicine Taipei Medical University Taipei Taiwan; ^5^ Cell Physiology and Molecular Image Research Center, Wan Fang Hospital Taipei Medical University Taipei Taiwan; ^6^ Research Center of Cancer Translational Medicine Taipei Medical University Taipei Taiwan; ^7^ Cancer Research Center Taipei Medical University Hospital Taipei Taiwan; ^8^ Cancer and Immunology Research Center National Yang Ming Chiao University Taipei Taiwan; ^9^ Department of Oncology Taipei Veterans General Hospital Taipei Taiwan

**Keywords:** CEA, CEA regulation, *CEACAM5*, colorectal cancer, DNA methylation

## Abstract

Colorectal cancer (CRC) is a globally common cancer, and the serum carcinoembryonic antigen (sCEA) is widely applied as a diagnostic and prognostic tumor marker in CRC. This study aimed to elucidate the mechanism of CEA expression and corresponding clinical features to improve prognostic assessments. In CRC cells, hypomethylation of the *CEACAM5* promoter enhanced CEA expression in HCT116 and HT29 cells with 5‐aza‐2′‐deoxycytidine (5‐Aza‐dC) treatment. Our clinical data indicated that 64.7% (101/156) of CRC patients had an sCEA level above the normal range, and 76.2% (77/101) of those patients showed a lower average CpG methylation level of the *CEACAM5* promoter. The methylation analysis showed that both CRC cell lines and patient samples shared the same critical methylation CpG regions at −200 to −500 and −1000 to −1400 bp of the *CEACAM5* promoter. Patients with hypermethylation of the *CEACAM5* promoter showed features of a *BRAF* mutation, *TGFB2* mutation, microsatellite instability‐high, and preference for right‐sided colorectal cancer and peritoneal seeding presentation that had a similar clinical character to the consensus molecular subtype 1 (CMS1) of colorectal cancer. Additionally, hypermethylation of the *CEACAM5* promoter combined with evaluated sCEA demonstrated the worst survival among the patients. Therefore, the methylation status of the *CEACAM5* promoter also served as an effective biomarker for assessing disease prognosis. Results indicated that DNA methylation is a major regulatory mechanism for CEA expression in colorectal cancer. Moreover, our data also highlighted that patients in a subgroup who escaped from inactivation by DNA methylation had distinct clinical and pathological features and the worst survival.

## Graphical abstract

This study investigated the regulatory mechanism of carcinoembryonic antigen (CEA) expression in colorectal cancer (CRC). The study found that DNA hypomethylation of the CEACAM5 promoter enhanced CEA expression in CRC. Furthermore, a subgroup of patients with a hypermethylated CEA promoter had molecular and clinical features similar to the consensus molecular subtype 1 (CMS1) of colorectal cancer and presented with a poor prognosis.

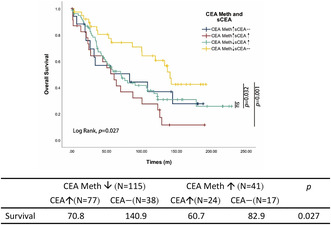

**Abbreviations**
CAMs: cell adhesion molecules
CIMP: CpG island methylator phenotype
CMS1: consensus molecular subtype 1
COSMIC: Catalogue of Somatic Mutations in Cancer
CRC: colorectal cancer
DNMT1: DNA methyltransferase 1
ICIs: immune checkpoint inhibitors
MSI: microsatellite instability,
MSI‐H: MSI‐high
RCC: right‐sided colon cancer
sCEA: serum carcinoembryonic antigen
TFs: transcription factors
Th1: type 1 helper T

## 1 INTRODUCTION

Colorectal cancer is the third most common cancer and the fourth leading cause of cancer deaths in the world.^1^ In Taiwan, CRC was the second most common cancer type and the third leading cause of cancer deaths in 2017.^2^ CRC has unique features such as several known genetic variations, genomic instability, and a CIMP.^3^, ^4^ CRC tumorigenesis is highly related to those genetic and epigenetic variations. Chromosomal instability and MSI are being researched to clarify the pathogenesis and prognosis of CRC. Aberrant DNA methylation, through the CIMP, enhances DNA hypermethylation at promoter CpG islands of tumor suppressor genes or other tumor‐related genes, leading to transcription inactivation and gene silencing.^5^ Therefore, the CIMP is considered an early event characteristic of the serrated pathway of colorectal tumorigenesis.^6^

In clinical practice, surgery is the primary treatment modality for CRC followed by radiation and chemotherapy, but advanced CRC still has poor survival outcomes. Therefore, in order to detect disease recurrence at an early stage, CRC surveillance is of utmost importance in clinical practice. This includes regular monitoring of images, such as imaging scans, as well as the utilization of valuable biomarkers. Early diagnosis through effective surveillance can significantly improve patient outcomes by enabling timely intervention and management of recurrent CRC. sCEA is a well known tumor marker for CRC, initially found on cell membranes of gastrointestinal tract cells during the embryonic period but decreasing after maturation.^7^ CEA has an immunoglobulin‐like structure and many glycosylation modification sites belonging to a group of CEA‐related CAMs (CEACAMs) containing 12 proteins (CEACAM1, ‐3 to ‐8, ‐16, ‐18 to ‐21).^8^ CEACAM1, CEACAM5, and CEACAM6 are the best characterized molecules in cancer processes and are considered valid clinical biomarkers and promising therapeutic targets in melanoma, lung, colorectal, and pancreatic cancers.^8^, ^9^, ^10^ In CRC, Gold and Freedman discovered CEA expression in colon cancer tissues that serves as a tumor marker in CRC.^11^ A clear correlation of tumor metastasis with sCEA in CRC was proven through in vivo and in vitro studies.^12^, ^13^ sCEA expression was correlated with the CRC prognosis and was mainly used for disease follow‐up and treatment response indicators.^14^ Serial measurements of sCEA are widely recommended in surveillance; however, agreement is lacking about what constitutes clinically significant changes in sCEA levels.^15^ Many clinical features have shown that the sCEA could predict the disease prognosis, severity of the disease, and response to therapy.^16^, ^17^ CRC patients with elevated sCEA tend to have the potential for liver metastasis, and the probability is highly correlated with the sCEA level. However, a previous contradictory survey indicated that ~30% of metastatic CRC cases did not have elevated sCEA.^18^, ^19^, ^20^ Moreover, the CEA expression regulatory mechanism during CRC progression is still unclear. Therefore, elucidating the CEA regulatory mechanism may improve the application of sCEA in clinical diagnoses.

Epigenetic regulation controls gene expressions through DNA methylation, histone modifications, and chromatin remodeling. Abnormal methylation changes of CpG islands of a tumor suppressor may be used as one of the available means for the early detection of tumor patients.^21^ Tran et al. first indicated the correlation of DNA hypomethylation with CEA expression in CRC cell lines.^22^ However, the sCEA level, DNA methylation pattern, and CRC's clinical characteristics were not addressed. To extend the understanding and improve sCEA practice in clinical applications, we conducted next‐generation sequencing (NGS)‐based methylation sequencing to profile the DNA methylation pattern of *CEACAM5* and analyze its correlations with clinical features in CRC samples.

## 2 MATERIALS AND METHODS

### 2.1 Cell lines

Two human CRC cell lines, HCT116 and HT29, were used in this study. The cell lines were obtained from the American Type Culture Collection (Manassas, VA, USA) and were authenticated before experiments were performed. These cells were cultured in Dulbecco's modified Eagle's medium (DMEM; Gibco/Life Technologies) supplemented with 10% fetal bovine serum (FBS, Gibco/Life Technologies) and 100 U/mL of penicillin. Cells were cultured at 37°C in a 5% CO_2_ incubator.

### 2.2 Clinical CRC samples

Surgically resected colon tissues with neoplastic and non‐neoplastic areas were obtained after CRC surgery. A sample was collected after a patient signed an informed consent form, and the protocol was approved by the institutional review board (IRB) of Taipei Veterans General Hospital (TVGH; IRB no. 2019‐01‐016BC). According to the pathology reports, the clinicopathological features of these samples and patients were collected in TVGH from August 2010 to May 2016. All subjects had been diagnosed with CRC. The diagnostic pathology reports were performed using paraffin‐embedded sections combined with an immunohistochemical (IHC) study of BRAF and MMR proteins to know the BRAF and MSI status. Tissues were submerged in an RNAlater solution (ThermoFisher Scientific) for further DNA extraction. Clinical CEA measurements were performed at the Department of Pathology and Laboratory Medicine of TVGH using an electrochemiluminescence immunoassay (ECLIA) method with an analytical sensitivity of 0.3 ng/mL.

### 2.3 5‐Aza‐2′‐deoxycytidine (5‐Aza‐dC) treatment of cell lines

The HCT116 and HT29 colon carcinoma cell lines were seeded the day before treatment and then treated with 5‐aza‐dC for 48 h. The treated concentration of 5‐aza‐dC was 10 μM for HCT116 and 15 μM for HT29 cells. After treatment, cells were harvested and washed with 1× phosphate‐buffered saline (PBS) before genomic RNA and DNA extraction for the CEA expression and DNA methylation surveys.

### 2.4 Extraction of DNA and quantification of CEA messenger (m)RNA

DNA from cell lines and colon tissues was extracted using a DNA extraction kit system (PicoPure DNA extraction kit, MDS Analytical Technologies) for the DNA methylation survey. Total RNA was extracted from cell lines using an RNeasy Mini kit (Qiagen). Total RNA (1 μg) was reverse‐transcribed with a high‐capacity RNA‐to‐complementary (c)DNA Kit (ThermoFisher Scientific). A real‐time reverse‐transcription polymerase chain reaction (PCR) was performed on a Roche LightCycler® 96 System using FastStart Essential DNA Green Master with the primers forward: CTGGCCGCAATAATTCCATAG and reverse: CCAGCTGAGAGACCAGGAGAA for CEA mRNA measurements.

### 2.5 Quantitative DNA methylation analyses by next‐generation sequencing

The promoter methylation status of *CEACAM5* was determined by NGS on bisulfite‐treated genomic DNA. Extracted DNA (100 ng) from the cell lines and clinical tissues was used for bisulfite treatment and methylation evaluation. A Zymo EZ DNA methylation Lightning Kit (Zymo Research) was used for bisulfite conversion, followed by an EpiGnome™ Kit (Epicenter) to prepare the bisulfite sequencing libraries before sequencing. Sequencing was performed with an Illumina HiSeq2500 genome sequencer (USA) to determine the reads of thymine and cytosine in each CpG site in the sequence. The proportion of reads between thymine and cytosine in the CpG sites can show the percentage of methylation of CpG sites. The methylation of CpG sites in the region from 1 to −1800 bp relative to the transcription start site of the *CEACAM5* promoter sequence was analyzed.

HCT116 DKO non‐methylated DNA and human HCT116 DKO methylated DNA in the Human Methylated and Non‐methylated DNA Set (Zymo Research) were respectively used as the positive and negative controls in the methylation survey. The methylation level was calculated from reads between the selected *CEACAM5* promoter sequence on each CpG site with reads of thymine and cytosine.

### 2.6 Mutation characteristic analysis

Mutation analyses of KRAS codons 12 and 13 and BRAF codon 600 were performed by pyrosequencing at the pathology department for clinical requirements. Genomic DNA was amplified by a PCR and sequenced with the PyroMark™ KRAS kit and the PyroMark™ BRAF kit according to the manufacturer's instructions. Results were part of cancer pathology reports and contained relevant information for clinical management.

Other colon cancer genetic mutation evaluations were performed by MassArray with hotspots reported by the COSMIC database, and 139 mutations in 12 genes were checked. The PCR for mutation detection was designed by MassArray Assay Design 3.1 software (Sequenom), and DNA products were analyzed by the MassArray Analyzer 4 system (Sequenom) and Typer 4.0 software (Sequenom) to detect mutations.

### 2.7 Statistical methods

Statistical analyses were performed using SPSS version 24.0 software (SPSS, Chicago, IL, USA). Data are shown as the prevalence, mean (standard deviation (SD)), or median (range). Discrete variables were compared using a chi‐squared test or Fischer's exact test as appropriate. Continuous variables were compared using the Mann–Whitney *U*‐test. The overall survival (OS) was displayed with Kaplan–Meier survival curves and compared using the log‐rank test. All statistical tests were two‐sided, with the threshold for significance set to *p* < 0.05.

## 3 RESULTS

### 3.1 Patient and tumor characteristics

This study included 156 Taiwanese CRC samples from the TPE‐VGH Biobank from August 2010 to May 2016. The clinical characteristics of the patient cohort are shown in Table 1. There were 64.7% (101/156) male patients and 35.3% (55/156) female patients, and the median age at diagnosis was 69 (range 35–92) years. Additionally, 26.3% (41/156) of the patients had a smoking history, while 73.7% (115/156) did not. The observed increase in sCEA levels shows no significant difference between the smoking group and the non‐smoking group (61% vs. 50.4%, *p* = 0.227). Among them, 23.1% (36/156) of patients were diagnosed with stage IV CRC and had received palliative primary tumor resection, among which 61.1% (22/36) of patients had liver metastasis, 27.8% (10/36) of patients had lung metastasis, and 19.4% (7/36) of patients had peritoneal seeding disease among these 36 stage IV CRC patients. The rest of the patients in stages I–III received surgical resection of the tumor lesion under curative intent. Figure S1 illustrates the adjuvant therapy and recurrence patterns in patients with stages I–III CRC. For stage I CRC, one patient underwent adjuvant chemotherapy due to an unclear resection margin. Of these patients, one out of 14 experienced lung metastasis during the follow‐up period. For stage II CRC, 10 patients received adjuvant chemotherapy based on high‐risk factors as per the National Comprehensive Cancer Network (NCCN) guidelines. Within this group, there were two instances of local recurrence, three of lung metastasis, and one of peritoneal seeding during the follow‐up. In stage III CRC, 79.3% of patients received adjuvant chemotherapy according to the NCCN guidelines. The remaining patients declined adjuvant chemotherapy due to personal reasons or poor health conditions. Among these stage III patients, nine developed liver metastasis, seven had peritoneal seeding, six had lung metastasis, three experienced local recurrence, and three had bone metastasis. The overall recurrence rate for stage III CRC in this study was 34.5%.

**TABLE 1:** Clinical characteristics of colorectal cancer patients.

Patient characteristics (*N* = 156)	
Age (range) years	69 (35–92)
Gender	
Male	101 (64.7%)
Female	55 (35.3%)
Smoking	41 (26.3%)

Tumor characteristics	
Stage	
I	14 (8.9%)
II	48 (30.8%)
III	58 (37.2%)
Recurrence	30 (25.0%)
Recurrent liver metastasis	9 (33.3%)
Recurrent peritoneal seeding	8 (29.6%)
IV	36 (23.1%)
Synchronous liver metastasis	22 (61.1%)
Synchronous peritoneal seeding	7 (19.4%)
Location	
Right‐sided	42 (26.9%)
Left‐sided	114 (73.1%)
Mucinous tumor	21 (13.5%)
Poor differentiation	11 (7.1%)
CEA (median, ng/mL)	7.83
≦5	55 (35.3%)
>5	101 (64.7%)
Genetic variants	
*KRAS* mut	42 (26.9%)
*BRAF* mut	7 (4.5%)
Loss of chromosome 18q	58 (37.2%)
*APC* mut	46 (29.5%)
*SMAD4* mut	9 (5.8%)
*TGFβ2* mut	4 (2.6%)
*TP53* mut	48 (30.8%)
*PIK3CA* mut	3 (1.9%)
*PTEN* mut	1 (0.6%)
*FBXW7* mut	17 (10.9%)
*HRAS* mut	1 (0.6%)
*NRAS* mut	13 (8.3%)
*AKT1* mut	3 (1.9%)
MSI‐H	20 (12.8%)

We next compared the genetic variants and clinical features of these primary tumors. Results indicated that 26.9% (42/156) of patients had a *KRAS* mutation, 4.5% (7/156) of patients had a *BRAF* mutation, 37.2% (58/156) of patients had loss of chromosome 18q, 29.5% (46/156) of patients had an *APC* mutation, 5.8% (9/156) of patients had a *SMAD4* mutation, 2.6% (4/156) of patients had a *TGFB2* mutation, 30.8% (48/156) of patients had a *Tp53* mutation, 1.9% (3/156) of patients had a *PIK3CA* mutation, 0.6% (1/156) of patients had a *PTEN* mutation, 10.9% (17/156) of patients had an *FBXW7* mutation, 0.6% (1/156) of patients had an *HRAS* mutation, 8.3% (13/156) of patients had an *NRAS* mutation, and 1.9% (3/156) of patients had an *AKT1* mutation. Furthermore, 12.8% (20/156) of patients had MSI‐H, 13.5% (21/156) of patients had mucinous tumors, 7.1% (11/156) of patients had poorly differentiated tumors, and 26.9% (42/156) of the CRC cases belonged to RCC in the overall population.

CEA is a cell surface glycoprotein used as a clinical biomarker for gastrointestinal cancers, especially colorectal malignancies. It promotes tumor development through its role as a cell adhesion molecule.^23^ An sCEA level of >5.0 ng/mL is considered positive.^24^ In these samples, 55 patients (35.3%) were categorized as normal (<5 ng/mL), and 101 patients (64.7%) had elevated sCEA levels of >5 ng*/*mL. The overall median sCEA level was 7.83 ng/mL. Since DNA methylation regulation is a critical epigenetic regulation in CRC progression and shows an inverse correlation with CEA expression,^22^ this implies that DNA methylation may control CEA expression. Although studies indicated that CEA expression was correlated with DNA hypomethylation in colorectal cell lines, the DNA methylation status of *CEACAM5* has not previously been evaluated in clinical samples. Therefore, we evaluated the *CEACAM5* promoter methylation status and sCEA expression among the different stages of CRC patients and CRC cell lines to improve our understanding of sCEA's role in diagnoses.

### 3.2 Survey of CEA levels and DNA methylation statuses in CRC cell lines

**FIGURE 1:**
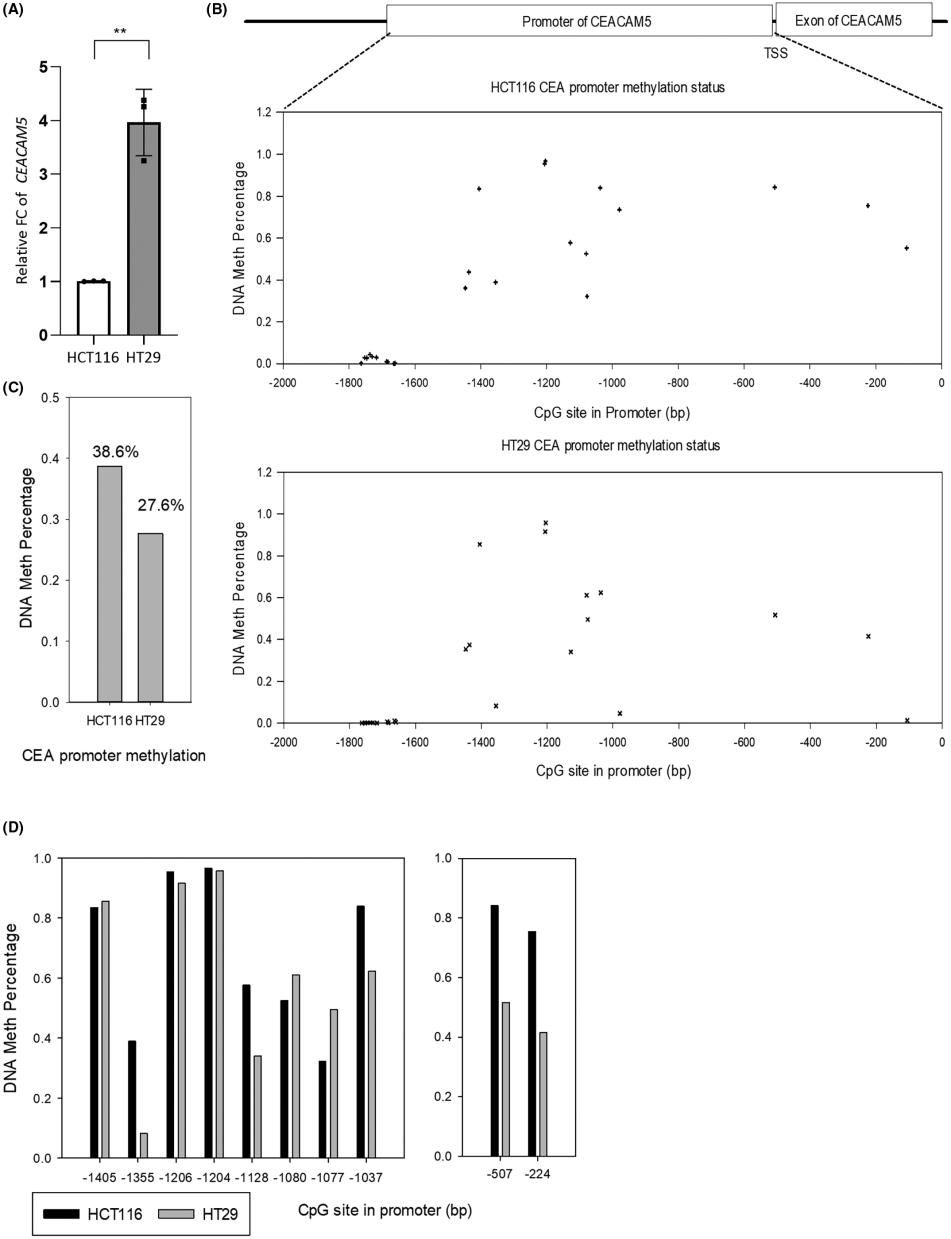
DNA methylation patterns of CpG sites in the *CEACAM5* promoter of colorectal cancer (CRC) cell lines. (A) RT‐qPCR for analyzing the relative expression of *CEACAM5* in HCT116 and HT29 cells. Data are presented as the mean ± SD. *n* = 3 independent experiments (each experiment contained two technical replicates). (B) NGS analysis results of CpG methylation distribution in the *CEACAM5* promoter of HCT116 and HT29 cells. (C) Average percentages of CpG methylation of the *CEACAM5* promoter in HCT116 and HT29 cells. (D) Average percentages of CpG methylation of HCT116 and HT29 cells in the promoter at −200 to −500 and −1000 to −1400 bp from the transition start site (TSS) of *CEACAM5*. **p < 0.01.

We first examined CEA expression in two CRC cell lines. Quantitative PCR (qPCR) results showed that the CEA messenger (m)RNA level was significantly higher in HT29 compared to HCT116 cells (*p* < 0.001; Figure 1A). Next, we analyzed the DNA methylation pattern of CpG sites in the *CEACAM5* promoter by NGS (Figure 1B). Results showed that methylation of the *CEACAM5* promoter of HT29 cells (27.6%) was lower than that of HCT116 cells (38.6%), which indicated that hypomethylation of the *CEACAM5* promoter may enhance CEA mRNA production in CRC cell lines (Figure 1C). Moreover, sequencing data indicated that two central regions of CpG sites, of −200 to −500 (two CpG sites) and −1000 to −1400 bp (eight CpG sites) in the *CEACAM5* promoter region, showed the greatest difference between the two cell lines (Figure 1B,D).

**FIGURE 2:**
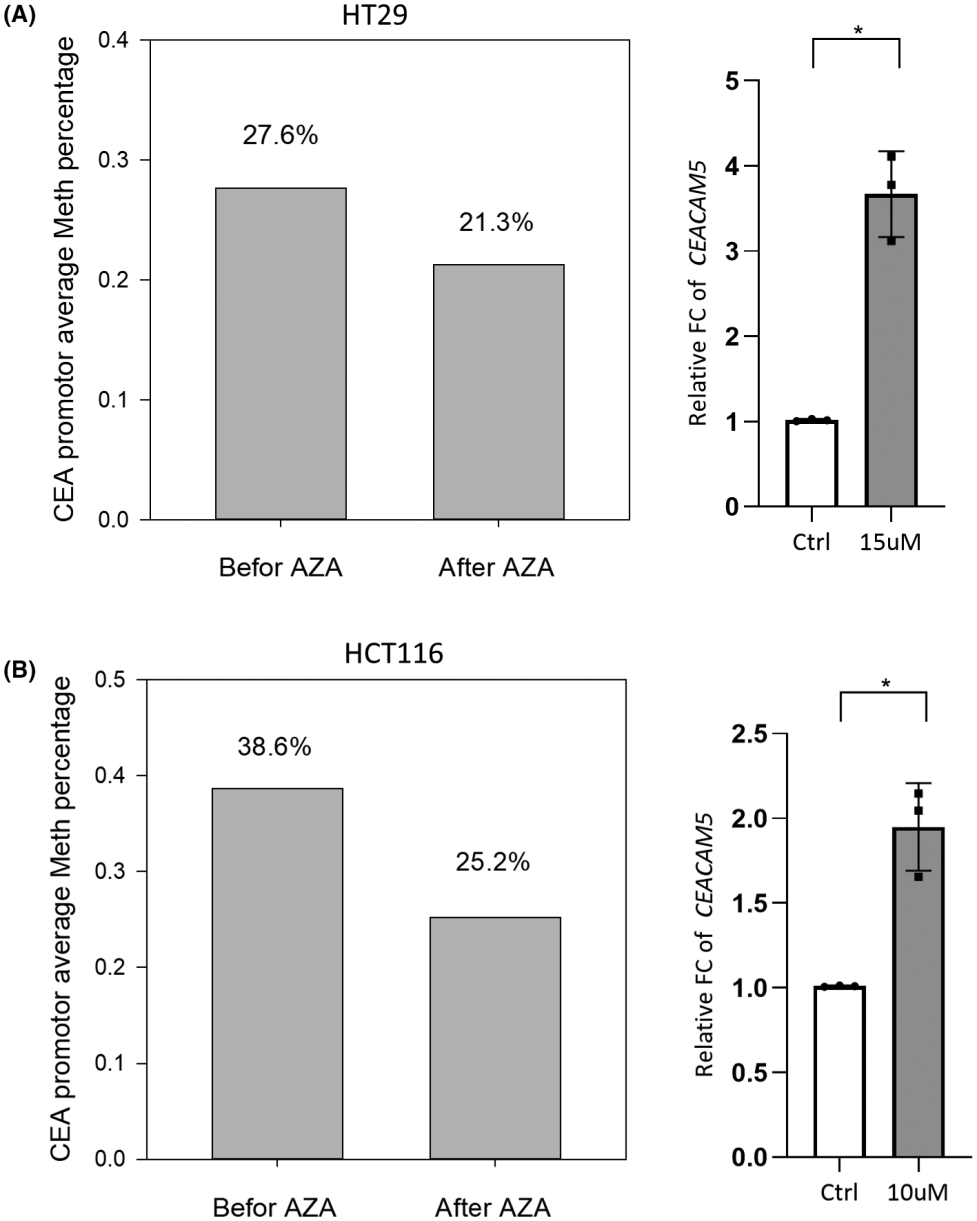
Effect of 5‐Aza‐dC on DNA methylation of the *CEACAM5* promoter and corresponding *CEACAM5* expression of colorectal cancer (CRC) cell lines. (A) Left: *CEACAM5* promoter methylation percentages before and after 5‐Aza‐dC treatment in HT29 cells. Right: Corresponding *CEACAM5* mRNA expressions in control and 5‐Aza‐dC‐treated cells. Data are presented as the mean ± SD. *n* = 3 independent experiments (each experiment contained two technical replicates). (B) Left: *CEACAM5* promoter methylation percentages before and after 5‐Aza‐dC treatment in HCT116 cells. Right: Corresponding *CEACAM5* mRNA expressions in control and 5‐Aza‐dC‐treated cells. Data are presented as the mean ± SD. *n* = 3 independent experiments (each experiment contained two technical replicates). *p < 0.05. See also Figures S2 and S3.

Next, we examined whether the DNA methylation status influences CEA expression. We treated CRC cells with 5‐aza‐2′‐deoxycytidine (5‐Aza‐dC). 5‐Aza‐dC is incorporated into nucleic acids and prevents methylation at CpG sites by irreversible covalent binding to DNMT1, leading to loss of methyltransferase activity and demethylation of DNA.^25^ Results indicated that, on average, methylated CEA promoters decreased from 27.6% to 21.3%, and the mRNA of *CEACAM5* increased in HT29 cells after 5‐Aza‐dC treatment (Figure 2A). Consistently, HCT116 cells treated with 5‐Aza‐dC showed that the average methylated promoter decreased from 38.6% to 25.2%, and CEA mRNA also increased (Figure 2B). Moreover, the NGS‐based methylation sequencing data indicated that the decrement in the methylated region in both 5‐Aza‐dC‐treated CRC cell lines occurred at −200 to −500 and −1000 to −1400 bp of the *CEACAM5* promoter region (Figures S2 and S3). These results indicated that the regions of −200 to −500 and −1000 to −1400 bp on the *CEACAM5* promoter are hot spots for DNA methylation changes.

### 3.3 Investigation of the *CEACAM5* methylation pattern in CRC tumors

**FIGURE 3:**
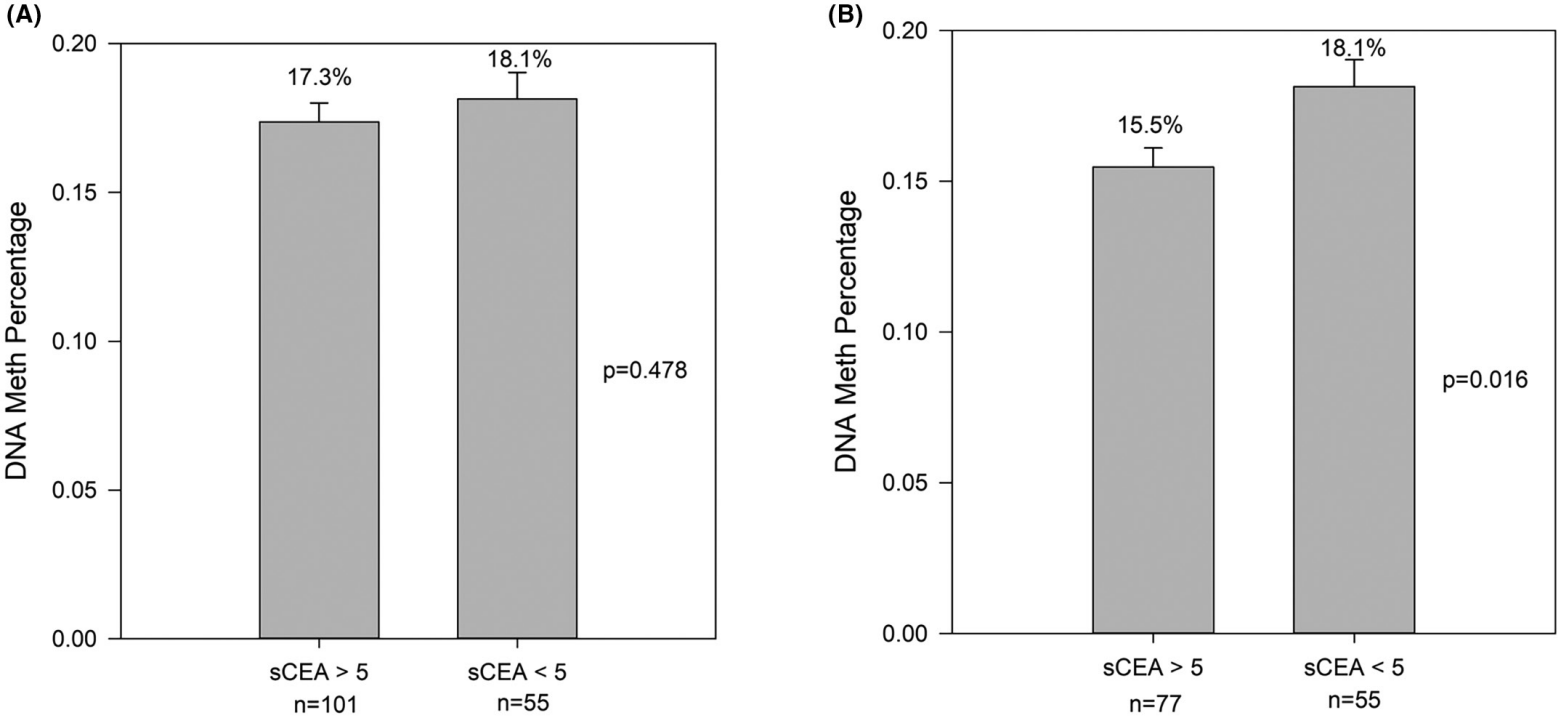
Serum carcinoembryonic antigen (sCEA) levels and corresponding DNA methylation percentages of the *CEACAM5* promoter. (A) The *CEACAM5* promoter methylation percentages of the sCEA at >5 ng/mL and <5 ng/mL. (B) *CEACAM5* promoter methylation percentages between the sCEA at >5 ng/mL and <5 ng/mL with the exclusion of 24 patients whose sCEA had escaped inactivation by DNA methylation. See also Tables 3 and 4.

We next examined the methylation status of the *CEACAM5* promoter in 156 CRC clinical samples and the corresponding adjacent normal tissues. Results indicated that 73.7% (115/156) of CRC tumors had decreased average CpG methylation over the *CEACAM5* promoter compared with the adjacent normal part, and 26.3% (41/156) of cases belonged to the increased *CEACAM5* methylation group (Table 2). Among the sCEA‐increased patients, 76.2% (77/101) of patients had lower CpG methylation of the *CEACAM5* promoter, and 23.8% (24/101) of patients had higher CpG methylation of the *CEACAM5* promoter (Tables 3 and 4). Although the tumor *CEACAM5* promoter methylation percentage in the elevated sCEA level group did not show significant difference compared with the normal sCEA group (*p* = 0.478; Figure 3A), when excluding 24 patients who escaped from inactivation by DNA methylation (those with increased *CEACAM5* promoter methylation and elevated sCEA), the increased sCEA expression group showed a significantly lower *CEACAM5* promoter methylation percentage (*p* = 0.016; Figure 3B). The relationship between tumor CEACAM5 promoter methylation and the corresponding sCEA can be seen in Figure S4. There was a borderline significant negative correlation between sCEA and CEACAM5 promoter methylation (*p* = 0.054). The results indicated that, in most CRC patients, increased sCEA expression is associated with a decrease in the methylation level of the *CEACAM5* promoter.

**TABLE 2:** Clinical characteristics between difference *CEACAM5* promotor methylation status of CRC patients.

	CEA Meth ↓ *N* = 115	CEA Meth ↑ *N* = 41	*p‐*value
Age	68.9	68.8	0.974
Gender male	64.3%	65.9%	0.862
*KRAS* mut	37.8%	37.5%	0.970
*BRAF* mut	0.9%	15%	0.001
Loss of 18q	40.0%	29.3%	0.222
*APC* mut	30.4%	26.8%	0.664
*SMAD4* mut	6.1%	4.9%	0.776
*TGFβ2* mut	0.9%	7.3%	0.025
*Tp53* mut	32.2%	26.8%	0.524
*PIK3CA* mut	15.6%	9.8%	0.352
*PTEN* mut	0.9%	0%	0.549
*FBXW7* mut	10.4%	12.2%	0.756
*HRAS* mut	0.9%	0%	0.549
*NRAS* mut	9.6%	4.9%	0.351
*AKT1* mut	0.9%	4.9%	0.109
MSI‐H	8.7%	24.4%	0.011
Right‐sided CRC	22.6%	39.0%	0.042
Mucinous	14.8%	9.8%	0.418
Poor differentiation	5.2%	12.2%	0.134
Stage IV	22.6%	24.4%	0.816
Synchronous liver metastasis	13.0%	17.1%	0.524
Synchronous peritoneal seeding	3.5%	7.3%	0.308
Recurrent liver metastasis	6.1%	4.9%	0.776
Recurrent peritoneal seeding	1.7%	14.6%	0.001
Survival	100.7 m	60.8 m	0.058

**TABLE 3:** Clinical characteristics between difference CEA level of CRC patients.

	CEA ↑ *N* = 101	CEA normal *N* = 55	*p‐*value
Age	69.2	68.4	0.771
Gender male	60.4%	72.7%	0.124
*KRAS* mut	38.6%	34.5%	0.615
*BRAF* mut	6.1%	1.9%	0.229
Loss of 18q	44.6%	23.6%	0.010
*APC* mut	29.7%	29.1%	0.936
*SMAD4* mut	5.0%	7.3%	0.552
*TGFβ2* mut	4.0%	0%	0.135
*Tp53* mut	32.7%	27.3%	0.485
*PIK3CA* mut	12.9%	16.4%	0.549
*PTEN* mut	1.0%	0%	0.459
*FBXW7* mut	9.9%	12.7%	0.588
*HRAS* mut	1.0%	0%	0.459
*NRAS* mut	6.9%	10.9%	0.390
*AKT1* mut	3.0%	0%	0.197
MSI‐H	13.9%	11.1%	0.627
Right‐sided CRC	29.7%	21.8%	0.283
Mucinous	14.9%	10.9%	0.491
Poor differentiation	6.9%	7.3%	0.936
Stage IV	29.7%	11.1%	0.001
Synchronous liver metastasis	19.8%	3.6%	0.006
Synchronous peritoneal seeding	5.9%	1.8%	0.870
Recurrent liver metastasis	7.9%	1.8%	0.118
Recurrent peritoneal seeding	4.0%	7.3%	0.370
Survival	60.4 m	137.1 m	0.023

**TABLE 4:** Clinical characteristics between CEACAM5 promotor methylation status of CRC patients with CEA elevated.

	sCEA ↑ Meth ↓ *N* = 77	sCEA ↑ Meth ↑ *N* = 24	*p‐*value
Age	68.8	70.3	0.574
Gender male	47 (61%)	14 (58.3%)	0.813
*KRAS* mut	30 (39%)	9 (37.5%)	0.898
*BRAF* mut	1 (1.3%)	5 (21.7%)	0.000
Loss of 18q	36 (46.8%)	9 (37.5%)	0.426
*APC* mut	24 (31.2%)	6 (25%)	0.564
*SMAD4* mut	5 (6.5%)	0	0.200
*TGFβ2* mut	1(1.3%)	3 (12.5%)	0.014
*Tp53* mut	25 (32.5%)	8 (33.3%)	0.937
*PIK3CA* mut	9 (11.7%)	4 (16.7%)	0.525
*PTEN* mut	1 (1.3%)	0	0.575
*FBXW7* mut	6 (7.8%)	4 (16.7%)	0.204
*HRAS* mut	1 (1.3%)	0	0.575
*NRAS* mut	6 (7.8%)	1 (4.2%)	0.541
*AKT1* mut	1 (1.3%)	2 (8.3%)	0.076
MSI‐H	6 (7.8%)	8 (33.3%)	0.002
Right‐sided CRC	18 (23.4%)	12 (50%)	0.037
Mucinous	11 (14.3%)	4 (16.7%)	0.775
Poor differentiation	3 (3.9%)	4 (16.7%)	0.031
Stage IV	24 (31.2%)	6 (25%)	0.444
Synchronous liver metastasis	15 (19.5%)	5 (20.8%)	0.885
Synchronous peritoneal seeding	3 (3.9%)	3 (12.5%)	0.119
Recurrent liver metastasis	6 (7.8%)	2 (8.3%)	0.932
Recurrent peritoneal seeding	1 (1.3%)	3 (12.5%)	0.014
Survival	70.8 m	60.7 m	0.196

**FIGURE 4:**
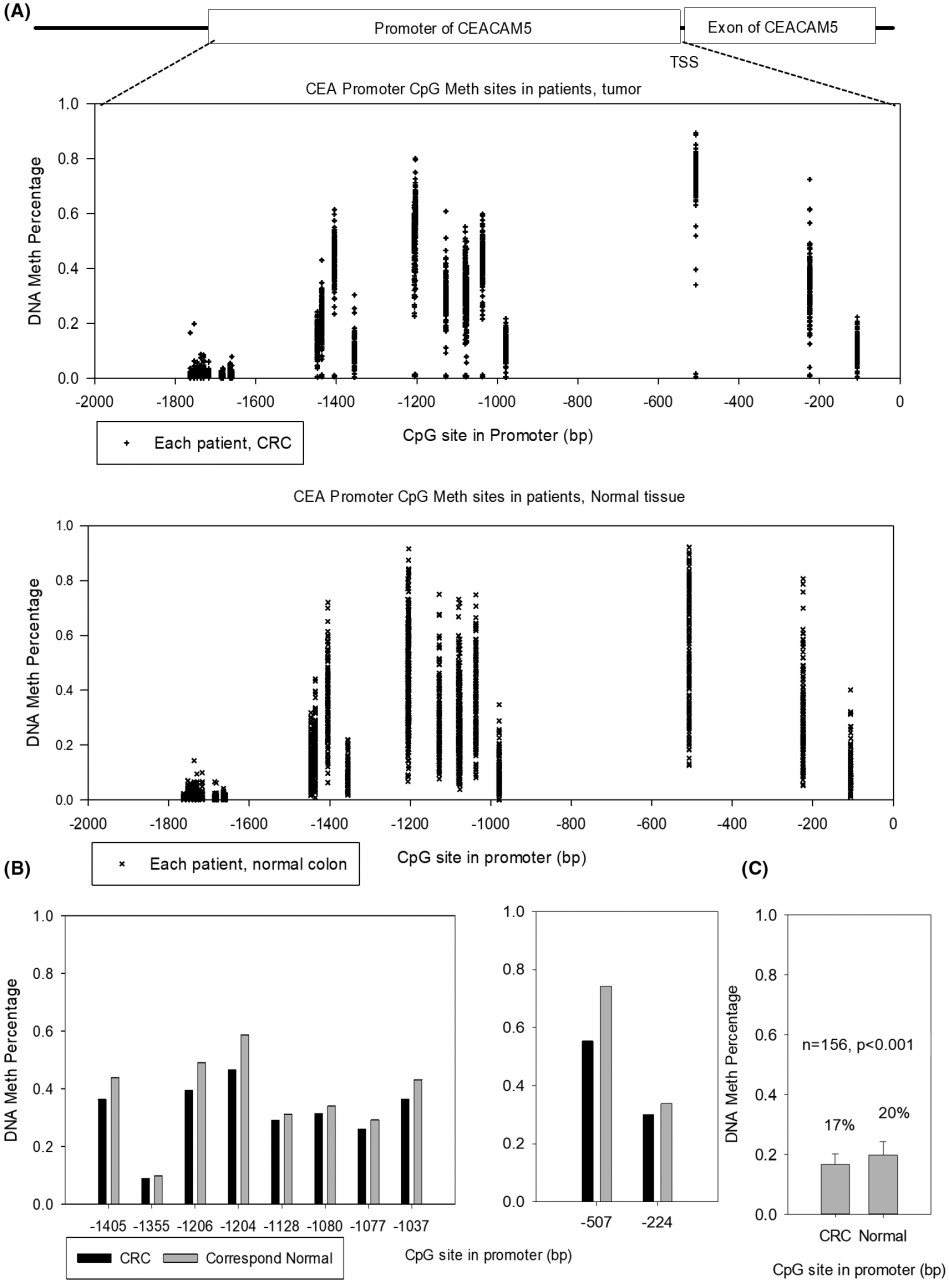
DNA methylation pattern of CpG sites in the *CEACAM5* promoter of colorectal cancer (CRC) patients. (A) NGS analytical results of the CpG methylation distribution of 156 CRC patients. (B) Average percentages of the CpG methylation pattern of the promoter at −200 to −500 and −1000 to −1400 bp from the TSS of *CEACAM5*. (C) Average CpG methylation percentages in 156 CRC patients. See also Figure S5.

Furthermore, DNA methylation profiling based on the NGS analysis of 156 patients indicated that the methylation difference in matched normal‐tumor pairs was located in regions −200 to −500 and −1000 to −1400 bp on the *CEACAM5* promoter (Figure 4A,B). This result was consistent with our finding of the DNA methylation hot spots in the CRC cell lines (Figures S2 and S3). Next, we compared the methylation ratio between the paired tumor and normal samples. Results showed that the average *CEACAM5* promoter methylation percentage in the tumor part was 17% ± 6%, whereas that of a matched adjacent normal part was 20% ± 3% (*p* < 0.001; Figure 4C). However, not all tumor parts had decreased *CEACAM5* promoter methylation levels than the paired normal samples. Interestingly, a consistent methylation pattern was noted in normal tissue in contrast to the divergence in the corresponding tumor part with lower methylation in the *CEACAM5* promoter. Cancer divergence was present in this survey of *CEACAM5* promoter methylation (Figure S5).

### 3.4 The clinical significance of sCEA level and *CEACAM5* promoter methylation in CRC patients

We analyzed the clinical features of patients who had increased or decreased *CEACAM5* promoter methylation and sCEA levels. Intriguingly, 26.3% (41/156) of patients with higher methylated *CEACAM5* promoter levels possessed clinical features such as a *BRAF* mutation, *TGFB2* mutation, MSI‐H, RCC, or recurrent peritoneal seeding compared to patients with lower *CEACAM5* methylated promoter levels (Table 2). In CRC cases with higher sCEA levels, clinical features showed a higher proportion of loss of the 18q mutation, more stage IV and synchronous liver metastasis, and poorer OS (Table 3). Among these patients, 23.8% (24/101) of them had higher methylation of the *CEACAM5* promoter accompanied by increased CEA expression, indicating that the CEA expression is not completely regulated by DNA methylation (Table 4). These patients exhibit features such as *BRAF* mutations, *TGFβ2* mutations, MSI‐H, right‐sided CRC, poor tumor differentiation, and recurrent peritoneal seeding. Notably, these features were not observed in the CEA elevation group. Those CRC patients also showed similar clinical features as the higher *CEACAM5* methylation group (Tables 2 and 4). Altogether the hypermethylated *CEACAM5* group showed these clinical features resemble the CMS1 classification of CRC with a *BRAF* mutation, *TGFB2* mutation, MSI‐H, and proximally located colon tumor.^4^

**FIGURE 5:**
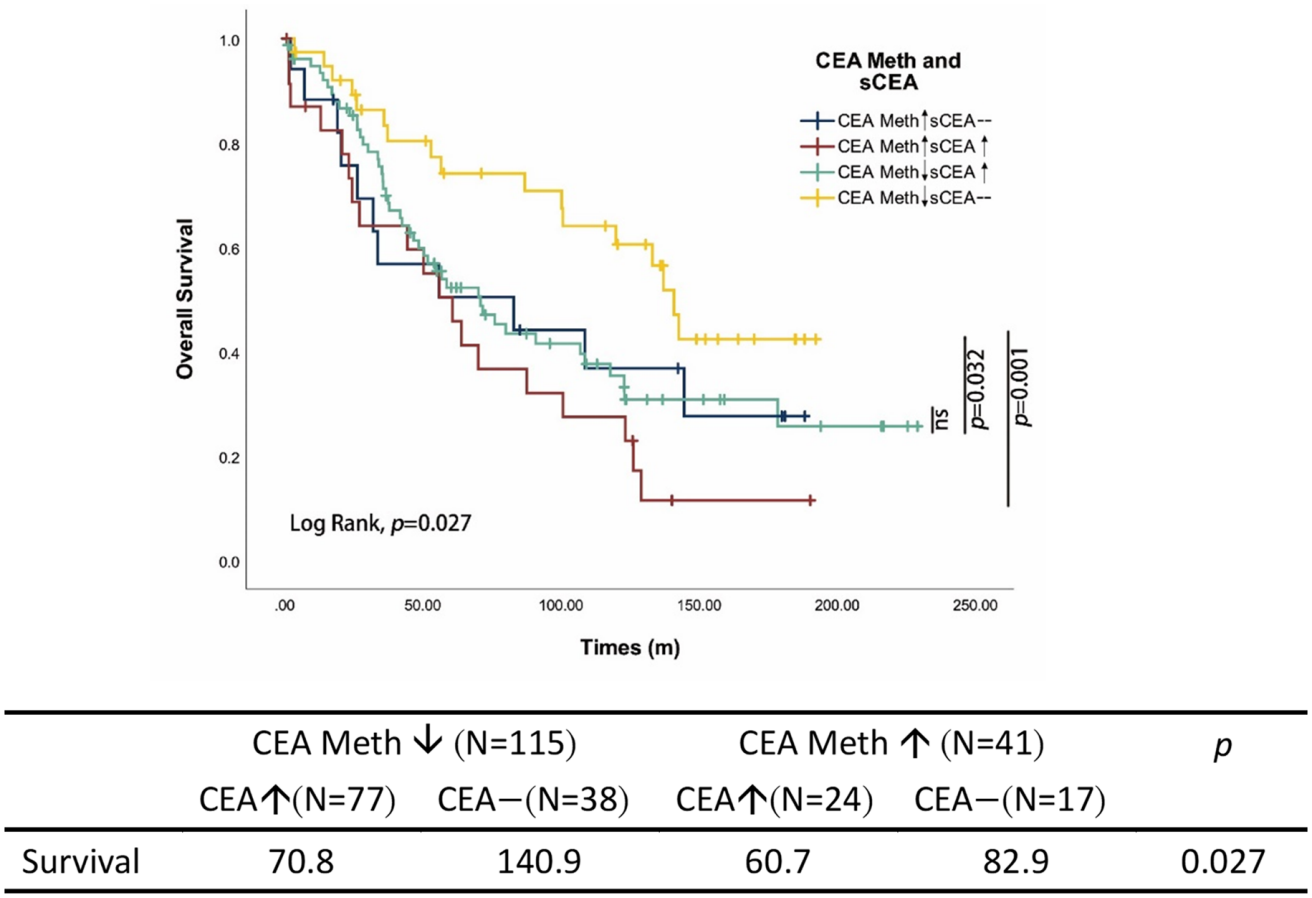
Kaplan–Meier curves of overall survival. The overall survival analysis in CRC patients according to *CEACAM5* promoter methylation status and sCEA level.

We next investigated the prognostic impact of sCEA combined with *CEACAM5* promoter methylation in CRC patients. The level of *CEACAM5* promotor methylation played a crucial role in determining the OS of CRC patients. The data revealed that the 24 patients who escaped sCEA inactivation by DNA methylation with hypermethylated *CEACAM5* promoter and elevated sCEA, demonstrated the worst OS. Conversely, patients with hypomethylated *CEACAM5* promoter and normal sCEA showed the best OS. There was no significant difference in the OS between patients with hypomethylated *CEACAM5* promoter and elevated sCEA and those with hypermethylated *CEACAM5* promoter and normal sCEA (Figure 5). The multivariable analysis, which considered factors such as age, gender, tumor location, *BRAF* mutation, MSI status, *KRAS* mutation, *TGFβ2* mutation, and poor tumor differentiation, indicated that the combination of CEACAM5 methylation and sCEA levels independently affected disease prognosis (Table 5, *p* = 0.023) The results indicated that integration of sCEA and *CEACAM5* promotor methylation presents a significant and informative approach for assessing disease prognosis.

**TABLE 5:** Univariate and multivariate analysis of factors influencing survival in colorectal cancer.

Variable	Univariate		Multivariate	
	HR (95% CI)	*p*‐value	HR (95% CI)	*p*‐value
Age	1.028 (1.009–1.048)	0.006	1.033 (1.012–1.055)	0.002
Gender				
Male	Ref.	0.654		
Female	0.907 (0.590–1.392)			
Location				
Right	Ref.	0.049	Ref.	0.061
Left	0.641 (0.411–0.999)		0.638 (0.398–1.021)	
*BRAF* mutation				
−	Ref.	0.435		
+	1.433 (0.581–3.537)			
MSI				
MSI‐H	Ref.			
MSS	0.806 (0.418–1.555)			
*KRAS* mutation		0.520		
−	Ref.			
+	1.307 (0.852–2.005)	0.219		
*TGFβ2* mutation				
−	Ref.	0.932		
+	1.052 (0.332–3.333)			
Poor differentiation				
−	Ref.	0.810		
+	0.895 (0.363–2.209)			
Meth/CEA		0.032		0.023
Meth↓/CEA‐‐	Ref.		Ref.	
Meth↑/CEA‐‐	1.799 (0.842–3.843)	0.130	2.040 (0.940–4.426)	0.071
Meth↓/CEA↑	1.837 (1.049–3.216)	0.033	1.960 (1.109–3.464)	0.021
Meth↑/CEA↑	2.684 (1.390–5.183)	0.003	2.980 (1.454–6.107)	0.003

## 4 DISCUSSION

CEA is a glycoprotein found by Gold and Freedman in colon cancer tissues, which was then applied as a CRC tumor marker.^26^ CEA is a cell membrane protein and can be cleaved by phospholipase C and phospholipase D and released into the circulation.^27^ sCEA expression was correlated with the CRC prognosis and was mainly used for disease follow‐up and as a treatment response indicator.^14^ In our study, most patients (64.7%, 101/156) had increased sCEA levels in the serum, and 76.2% (77/101) of them had a DNA hypomethylated *CEACAM5* promoter. However, not all CRC patients showed increased sCEA expression. Our data indicated that 35.3% (55/156) of CRC patients in our study presented with a normal sCEA level, and 16.6% (6/36) of stage IV CRC patients did not have elevated sCEA. Our previous study also indicated that 25.6% of stage IV CRC patients did not have elevated sCEA.^18^ Consistently, previous surveys showed that around 30% of metastatic CRC cases had no elevated sCEA.^19^, ^20^ A recent study indicated that tissue (t)CEA expression rather than sCEA is an independent factor associated with a poorer CRC prognosis in stages I–III of CRC.^28^ However, the post‐translational process of releasing sCEA and the effect of DNA methylation regulation on tCEA remain unresolved.

In this study, we evaluated the influence of DNA methylation on regulating sCEA expression. Our data indicated that the sCEA level was mainly regulated by DNA methylation control of the *CEACAM5* promoter. Traditionally, epigenetic reprogramming in cancer contributes to cancer development by directly inhibiting gene expressions through promoter hypermethylation or modification, particularly TFs.^29^ Therefore, mining associated TFs that can bind to these methylation hot spots and examining whether critical TFs are lost may explain why those patients had hypomethylation but low sCEA expression. Individual differences in those critical TFs may cooperate with DNA hypomethylation and regulate CEA expression within tumor samples. Interestingly, a subgroup of CRC patients had increased sCEA expression and had escaped from canonical regulation of gene inactivation by DNA methylation. Emerging studies showed that promoter hypermethylation is associated with gene activation through hypermethylation‐induced transcriptional activation.^30^ These data indicated that epigenetic contributions to transcriptional regulation occur in a more complex and more dynamic manner. However, the molecular mechanism of hypermethylation‐induced gene activation is currently unclear. Specific TFs and hypermethylation enhance gene activation under specific contexts showing that a more detailed investigation of the complex epigenetic regulation is warranted.

Moreover, our data indicated that the hypermethylated *CEACAM5* group showed molecular pathological features with a *BRAF* mutation, *TGFB2* mutation, MSI‐H, and proximally located colon tumors that were similar to CMS1 tumors. CMS1 tumors are enriched with activated Th1 lymphocytes, cytotoxic T cells, natural killer (NK) cell infiltration, and upregulated immune checkpoints such as programmed death ligand (PD)‐1. Therefore, CMS1 CRC patients could benefit from ICIs.^31^, ^32^ Whether tumors with hypermethylated *CEACAM5* share the same clinical characteristics as CMS1 tumors warrants further investigation. In clinical applications, previous studies have examined genetic methylation in CRC, identifying several genes with methylation patterns that serve as markers for CRC.^33^ Our data demonstrates that combining the assessment of *CEACAM5* promoter methylation status with sCEA levels provides a more comprehensive understanding of disease prognosis, highlighting the potential utility of *CEACAM5* promoter methylation level as a marker in clinical settings. Our study reveals that CRC patients with increased *CEACAM5* promoter methylation and elevated sCEA levels exhibit the poorest prognosis (Figure 5). However, the precise molecular mechanism through which hypermethylation induces gene activation, including the specific activation of the *CEACAM5* gene and its clinical correlation with CRC prognosis, remains unclear. It is hypothesized that hypermethylation may impede the binding of repressive TFs and distal regulatory elements.^30^, ^34^ Interestingly, hypermethylation‐induced gene activation has been observed in various contexts, such as induced pluripotent stem cells (iPSCs), early development, and malignancy. This raises the question of whether DNA methylation, as a potential novel pathway, instigates gene expression changes that drive malignancies to adopt a more pluripotent phenotype. In our study, this CRC subgroup displaying this trend toward a higher frequency of *BRAF* mutations (21.7%) and *TGFB2* mutations (12.5%) consisted of predominantly right‐sided CRC cases (50%) and exhibited a higher risk of recurrent peritoneal seeding (12.5%). All these factors have been associated with a poor prognosis for CRC. Based on our data, it can be inferred that hypermethylation accompanied by CEA activation signifies the worst clinical outcome among these patients. Those with a poor prognosis require more aggressive monitoring for recurrent disease in stages I–III for CRC, especially for peritoneal seeding, and a more intensive treatment approach in stage IV disease.

In conclusion, DNA methylation is the major regulatory mechanism governing sCEA expression in CRC, and hypomethylation could enhance sCEA expression. Furthermore, our data also identified two central regions of CpG sites at −200 to −500 and −1000 to −1400 bp in the *CEACAM5* promoter region, which are vital for regulating sCEA expression. Moreover, a subgroup of patients with hypermethylated *CEACAM5* promoters that escape from inactivation by DNA methylation demonstrated the molecular and clinical features with a *BRAF* mutation, *TGFB2* mutation, MSI‐H, recurrent peritoneal seeding, and worst prognosis, which may provide new insights into CRC.

## AUTHOR CONTRIBUTIONS

**Sheng‐Chieh Huang:** Data curation; formal analysis; funding acquisition; writing – original draft. **Shih‐Ching Chang:** Conceptualization; data curation; methodology. **Tsai‐Tsen Liao:** Data curation; formal analysis; funding acquisition; writing – review and editing. **Muh‐Hwa Yang:** Conceptualization; funding acquisition; project administration; writing – review and editing.

## FUNDING INFORMATION

This work was financially supported by TMU Research Center of Cancer Translational Medicine (DP2‐111‐21121‐01‐C‐03‐02 to T.T.L.) and NYCU Cancer Progression Research Center and Cancer & Immunology Research Center (to M.H.Y.) from The Featured Areas Research Center Program within the framework of the Higher Education Sprout Project by the Ministry of Education; National Science and Technology Council (NSTC 111‐2320‐B‐A49‐007 to M.H.Y., MOST 111‐2636‐B‐038‐004 to T.T.L., and MOST 110‐2221‐E‐075‐003‐MY3 to S.C.H.); National Health Research Institutes (NHRI‐EX109‐10919BI to M.H.Y.) and Taipei Veterans General Hospital (V112C‐130 and V112E‐002‐2 to M.H.Y. and V112C‐155 to S.C.H.).

## CONFLICT OF INTEREST STATEMENT

There is no conflict of interest in this study.

## ETHICS STATEMENT

Approval of the research protocol by an Institutional Reviewer Board: This study was approved by the Institutional Review Board of Taipei Veterans General Hospital (TPEVGH IRB no. 2019–01‐016 BC) and was performed in accordance with the Declaration of Helsinki.

Informed Consent: N/A.

Registry and the Registration No. of the study/trial: N/A.

Animal Studies: N/A.

## Supporting information


Figure S1.

Figure S2.

Figure S3.

Figure S4.

Figure S5.
