# Supplementary figures and images for: Detection and clinical significance of CEACAM5 methylation in colorectal cancer patients
Source: Cancer Sci. 2023 Nov 9;115(1):270–82. doi: 10.1111/cas.16012 (PMC10823287; doi:10.1111/cas.16012)

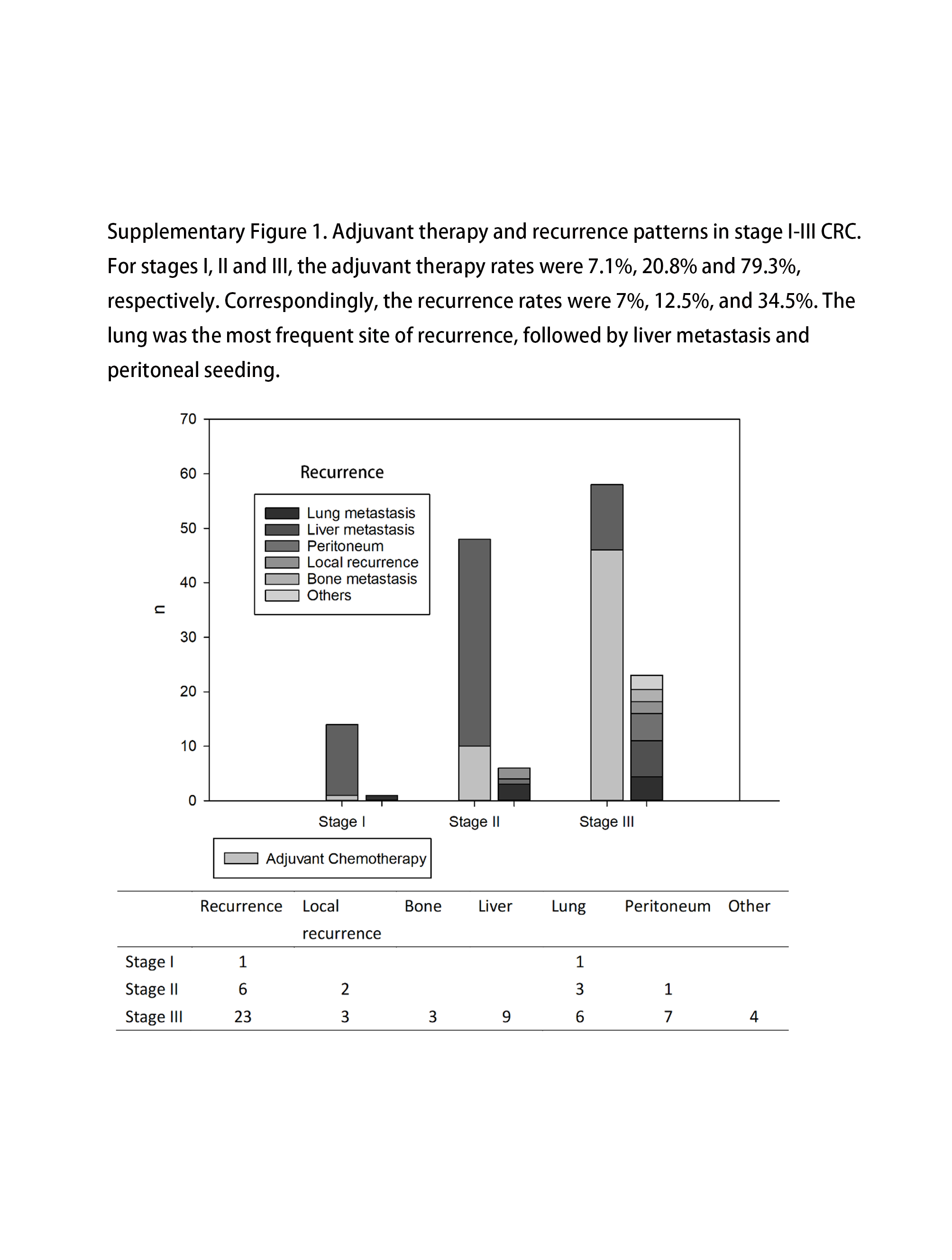

Supplement: Supplementary file 1 — Figure S1. [file CAS-115-270-s002.tif]

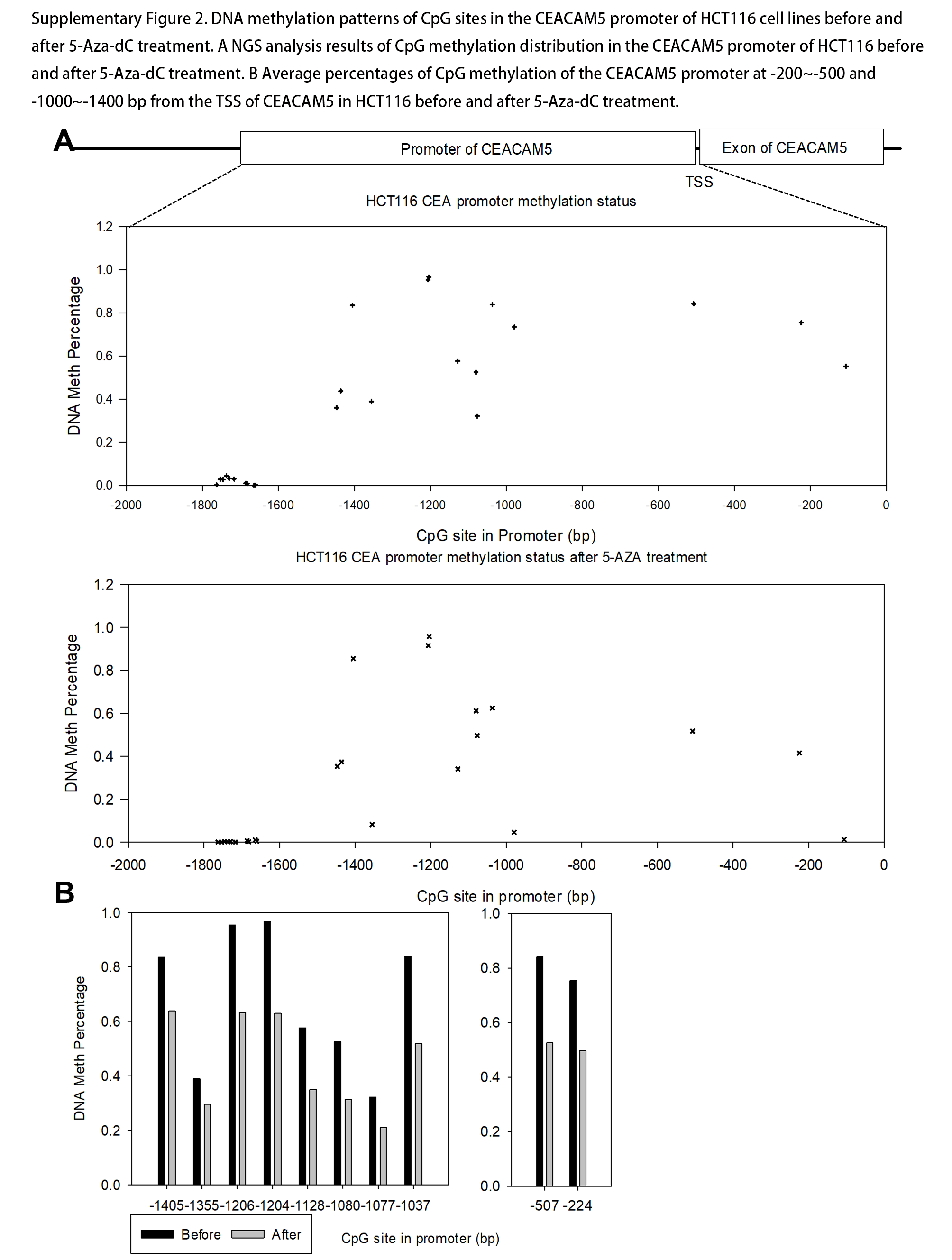

Supplement: Supplementary file 2 — Figure S2. [file CAS-115-270-s003.tif]

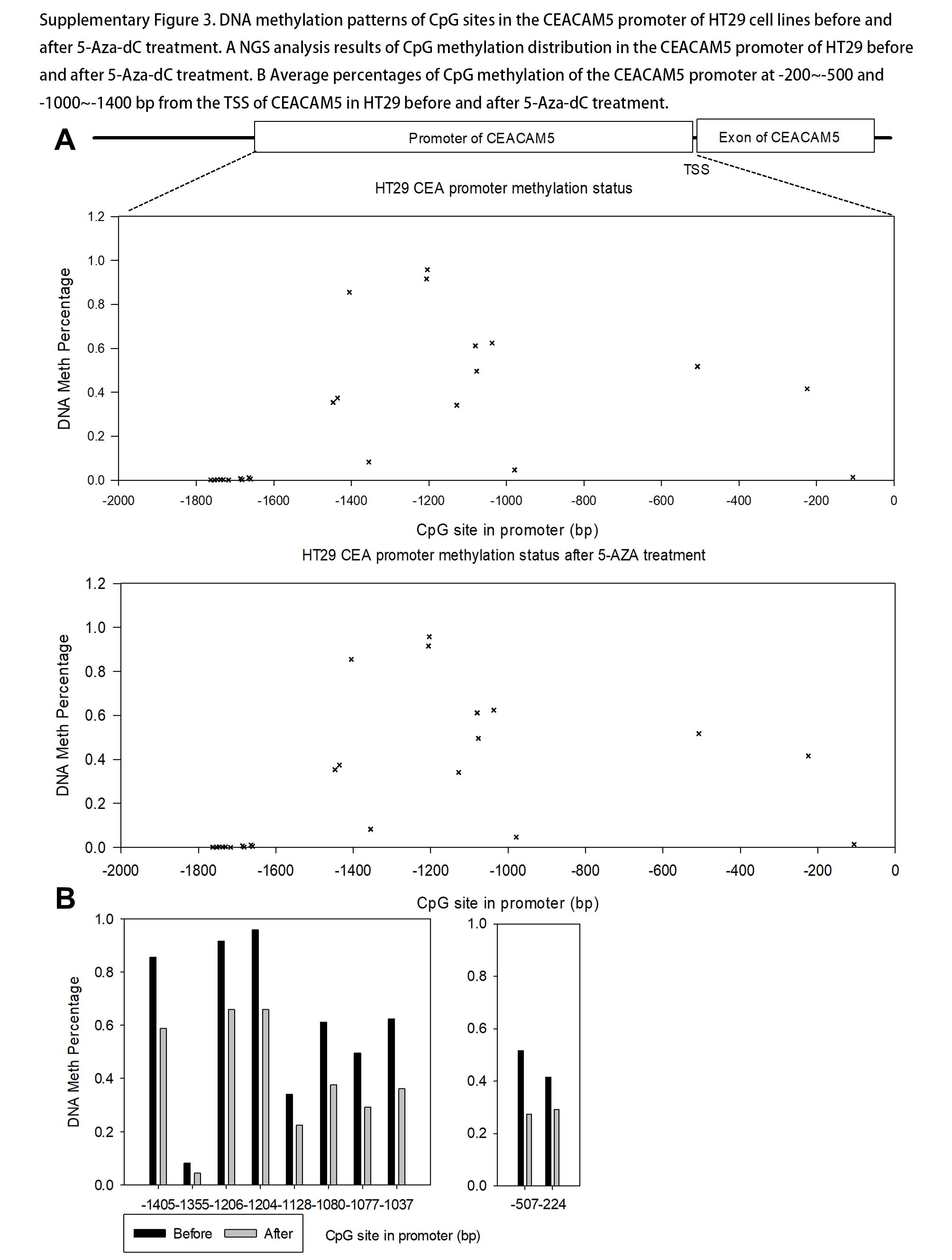

Supplement: Supplementary file 3 — Figure S3. [file CAS-115-270-s004.tif]

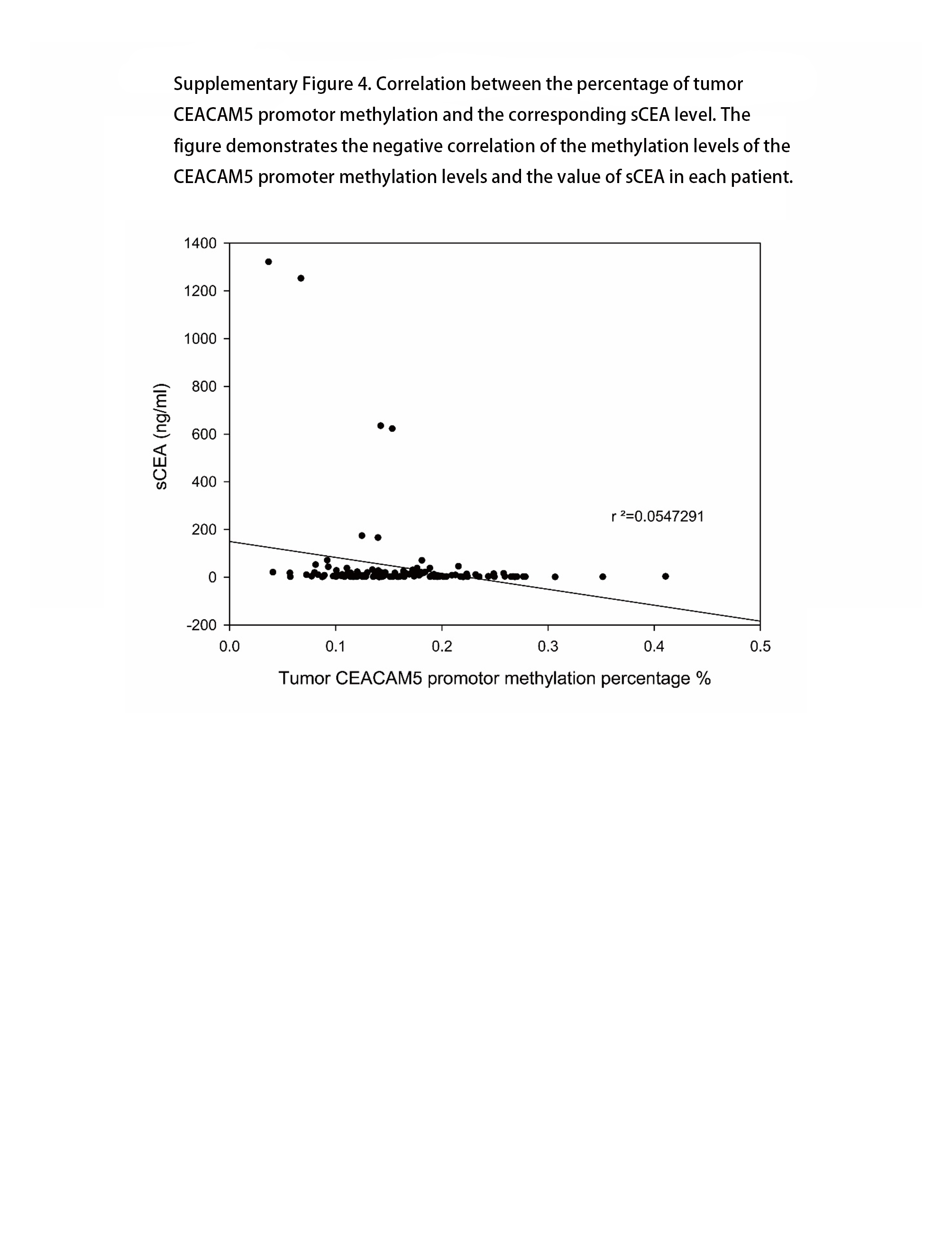

Supplement: Supplementary file 4 — Figure S4. [file CAS-115-270-s005.tif]

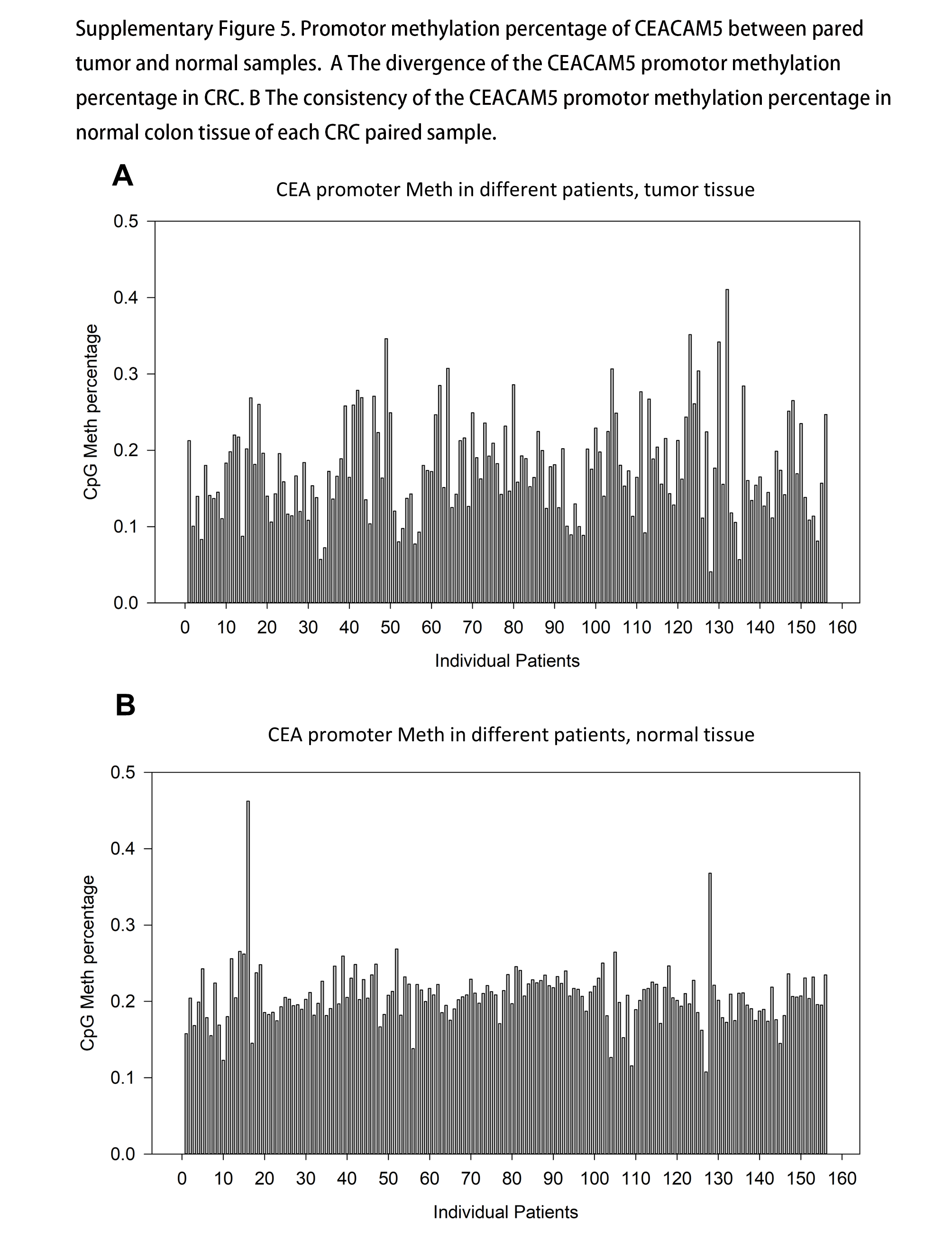

Supplement: Supplementary file 5 — Figure S5. [file CAS-115-270-s001.tif]
